# Effect and safety of immune checkpoint inhibitors in metastatic lung cancer: A retrospective study

**DOI:** 10.6026/973206300210534

**Published:** 2025-03-31

**Authors:** Arvind Kumar, Arun Raja, Dipika Narayan

**Affiliations:** 1 Department of General Medicine & Medical oncology, Near Sudha Dairy, Bokaro General Hospital, Biada Housing Colony, Ritudih, Bokaro Steel City, Jharkhand - 827012, India; 2 Department of General Medicine, Bokaro General Hospital, Bokaro Steel City - 827004, Jharkhand, India

**Keywords:** Non-Small Cell Lung Cancer, immunotherapy, nivolumab, real-life, efficacy

## Abstract

Cancer is a major global public health issue. Immune checkpoint inhibitors are increasingly used for managing advanced malignancies. However, their effect is limited by immune-related adverse events. Hence, a retrospective, single-institutional study found a 60% clinical benefit ratio among 30 patients receiving Immune checkpoint inhibitors therapy. Nonetheless, a small sample size, patient heterogeneity and retrospective design require further validation for more conclusive results.

## Background:

Immune checkpoint inhibitors (ICIs), such as pembrolizumab and nivolumab, have revolutionized the treatment of metastatic lung cancer, particularly non-small cell lung cancer (NSCLC). By targeting immune checkpoints like PD-1/PD-L1 and CTLA-4, these therapies enhance the body's immune response against tumor cells, offering durable responses and improved survival in a subset of patients. Their efficacy has been demonstrated in several clinical trials, showing significant improvement in overall survival compared to traditional chemotherapy. However, while ICIs offer promising therapeutic benefits, their use are associated with immune-related adverse events, which can affect various organs and require careful management. Understanding the balance between therapeutic efficacy and safety remains critical for optimizing patient's outcomes in metastatic lung cancer. According to international agency for research on cancer, this trend is probably caused by the increasing prevalence of cancer types with poorer prognoses as well as the lack of prompt access to diagnosis and treatment [1]. Men possess a 7.34% and females a 6.28% cancer mortality risk before the age of 75 [2].

## Materials and Methods:

## Study site:

Department of Medical Oncology, Apollo Multispecialty Hospital.58, Canal Circular Road Kolkata - 700054

## Study population:

Study population was the patients diagnosed with advanced stage cancer recent as well as old who is receiving immune check point inhibitors in Medical oncology department. All those patients who received immunotherapy from Jan 2018 to June 2021 were studied.

## Study design:

A Retrospective, single institutional, Observational study".

Sample size 30 patients

## Sampling method:

Non-Probability sampling

## Study period:

Recruitment time-from February 2020 to June 2021 (all those patients who received immunotherapy from Jan 2018 to June 2021 was studied) Analysis of data from august 2021 to November 2021.

## Inclusion criteria:

Advance solid cancer patients all with Performance Status 1 for the Eastern Cooperative Oncology Group (ECOG) or 3 who received ICIs after the failure of chemotherapy at our center or chemotherapy naive cases who were eligible to receive immune checkpoint inhibitors.

## Exclusion criteria:

[1] Performance status Eastern Cooperative Oncology Group 4.

[2] Patients who got therapy for fewer than four weeks were not included in the study.

[3] Hepatitis B- and hepatitis C-infected patients or carriers.

[4] Patients diagnosed with Ulcerative colitis.

[5] Prior auto immune disease on medications.

[6] Solid organ Transplant patients on immunosuppressive agents.

[7] Patients unwilling to give consent.

[8] Unable or unwilling to as determined by the investigator, abide with the protocol's guidelines.

[9] Concurrent enrolment in another clinical trial.

## Result and analysis:

## Results:

In Table 1 Cancer remains a significant global health concern, with approximately 18.1 million new cases and 9.6 million cancer-related deaths reported worldwide. According to estimates from the International Agency for Research on Cancer (IARC), the lifetime risk of developing cancer is substantial, affecting approximately 1 in 5 men and 1 in 3 women. Additionally, the mortality burden is considerable, with 1 in 8 men and 1 in 11 women succumbing to the disease. In recent years, immune checkpoint inhibitors (ICPIs) have emerged as a promising therapeutic approach for managing advanced malignancies. These agents work by enhancing the immune system's ability to recognize and attack cancer cells. However, despite their clinical benefits, the overall effectiveness of Immune checkpoint inhibitors is constrained by immune-related adverse events (irAEs), which can lead to significant morbidity and even limit the continuation of therapy. A retrospective, single-institutional study conducted at Apollo Multispecialty Hospital in Kolkata between 2018 and 2021 evaluated the clinical outcomes of Immune checkpoint inhibitors therapy in 30 patients with advanced cancers. The study reported a clinical benefit ratio of 60%, indicating a positive therapeutic response in a majority of patients. However, several limitations were noted, including the small sample size, heterogeneity of the patient population and the retrospective nature of the study. These factors limit the generalizability of the findings and highlight the need for larger, well-controlled prospective studies to further validate the efficacy and safety of Immune checkpoint inhibitors in diverse patient cohorts.

## Discussion:

In this investigation, 30 patient records in all were looked at. The bulk of the patients (56.7%) were in the 50 to 60 age range. The average age was 54. The median age in this study was lower than the median ages in the majority of other investigations, which ranged from 62 to 64 years. This discrepancy might be caused by the fact that we chose study participants who were physically fitter and had higher performance status. In terms of gender distribution, this study included a 20% female population and an 80% male population. Similar to other studies, this one also includes a sizable male population. This might be because men are more likely than women to get cancer and because men present to medical facilities for additional treatment earlier than women do. In this study, comorbid conditions affected 87% of participants, whereas only 23% were comorbidity-free. Most of the participants had diabetes, hypertension and ischemic heart disease. 33.3 percent of the trial participants had diabetes or hypertension. Only 13% and 23% of persons, respectively, had diabetes and hypertension. The relevance of our findings could not be justified because comorbidities were not considered in many earlier studies [3]. The cohort consisted of two patients with nasopharyngeal cancer (nasopharyngeal cancer), nineteen Having bladder cancer, three with lung cancer that is not small cell (Non-Small Cell Lung Cancer) and six with Root Canal Configuration or Renal Cell Carcinoma: kidney cancer. The majority of patients in our analysis had lung cancer, which was followed by renal cell carcinoma; this could be attributed to the fact that immunological check point inhibitors were first approved for use in these tumors rather than other types of malignancies; this is consistent with numerous other studies carried out globally over time. Sixteen patients (20%) had clear cell histology, two (6.7%) had the histology of squamous cell carcinoma and the final three (10%) had Urocellular cancer histology. Nineteen patients (63%) had adenocarcinoma histology. This also agrees with previously published findings. Adenocarcinomas were more common in this study because lung cancer was the primary diagnosis in the majority of our cases. All of the participants in this study had advanced metastatic cancer and each case's unique metastatic burden was evaluated. Lymph nodes, the liver, lung, bone and the brain were where mets were most frequently discovered. Metastasis in more than two organs was common. It suggests that a high proportion of cases involved multiple organ sites and that immunological check point inhibitors were effectively supplied to this patient population [4]. In this study, two immune checkpoint inhibitors were investigated: pembrolizumab and nivolumab. Only 20% of the patients received nivolumab; the bulk of them were given pembrolizumab. This might be as a result of the approval of pembrolizumab as a first-line therapy and promising results in lung and kidney cancer. There were a few patients who also received nivolumab and there was no difference in compliance between the two novel medicines. Additionally, compared to the other earlier research, both medications had greater tolerability and these innovative compounds could be used easily. Everyone received their prescription on schedule, with the exception of those who suffered grade-2 toxicity and there were no apparent treatment gaps [5]. Unfavorable immune-related events (IAEs), which can influence any organ, system and can occur either during the course of immunotherapy or after the course of treatment has ended, have been linked to immunotherapy and are new side effects for cancer patients. A study was conducted to examine the pattern of negative effects in individuals who received immunological check point inhibitors. Each visit included the recording and analysis of skin, gastrointestinal, endocrine and hepatic toxicity. Immune check point inhibitor dosages were also scheduled. Most skin-related negative effects that were mentioned were grade 1 issues. The majority of the grade 1 side effects, which affected 26 patients in total, were rashes; no additional skin damage was observed. After 10 cycles of pembrolizumab, only one patient developed a grade 2 adverse event, which gradually disappeared with timely treatment in accordance with the guidelines. Three study participants didn't encounter any toxicity at all over the entire study. Without any therapy, all of the grade 1 side effects subsided over time. This outcome is essentially consistent with all retrospective investigations using immunological check point inhibitors that have been published in the past. Most topical emollients and moisturizers were used to treat the symptoms; neither enteral nor topical steroids were administered [6]. Immunocheck point inhibitor medication did not result in any injury to the nervous system, the kidneys, or the blood. According to a large body of further research and case series, the bulk of the neurological side effects have been linked to the use of dual immune checkpoint inhibitors like nivolumab and ipilumab in combination.

An inflammation of the lung parenchyma known as pneumonitis is frequently found by computed tomography imaging. There are no pathognomonic clinical, pathologic, or radiological features of pneumonitis, despite the possibility of new or worsening cough, shortness of breath, increased oxygen need, chest pain and/or fever as presenting symptoms associated with immune therapy-induced pneumonitis. In 26 patients, pnemonitis was a grade 1 adverse impact; in 4, it was a grade 2 adverse effect. Due to the widespread Covid-19 infection at the time and the fact that no patients received steroids and all were treated alone with symptomatic care, a high prevalence of grade 1 toxicity was seen. Immune checkpoint inhibitors were not given until the grade 1 adverse impact subsided and in all 4 cases, steroid use was seen. Four people had grade 2 toxicity. Before starting immune checkpoint inhibitors after a brief gap, they all had chest imaging. This result was consistent with other trials using immunological check point inhibitors that have been previously reported [7]. Immune checkpoint therapy presents a special clinical difficulty in that patient with sometimes vague symptoms or complex aberrant test results must be evaluated for endocrine dysfunction. In this study, grade 1 toxicity was shown to have an effect on the thyroid gland; practically all cases of grade 1 toxicity were associated with primary hypothyroidism and 26 of these patients continued to take immunological check point inhibitors. Four people had Grade 2 toxicity, which was present. A hormone replacement dosage was used to treat every case of grade 2 toxicity. Seven patients were treated with whole-brain radiation treatment for grade 1 hypophysitis. Most cases of grade 1 hypophysitis were treated conservatively and as long as daily living activities were continued, everyone saw clinical recovery after a few months. An endocrine opinion is acquired in every case of hypophysitis and steroids are tapered off after a two to three week period. The results also matched those of earlier retrospective study [8]. Immunological check point therapy had GI adverse effects including colitis, hepatitis, gastritis and enterocolitis. Colitis was the only Grade 1 adverse effect that occurred in 8 research participants; all patients recovered after getting symptomatic therapy. Only three patients experienced grade 1 liver damage and all three of them recovered with symptomatic treatment. These outcomes are in line with earlier research. In the remaining members of the cohort, no additional treatment pauses or dosage reductions were necessary. Having no progression after three months of treatment constitutes a therapeutic benefit seen in 18/30 patients who underwent therapy for over three months, yielding a clinical benefit ratio of 60%. The 30 patients who were assessed for response did not experience complete remission; instead, 3 had partial response (response rate of 10%), 18 had stable disease (response rate of 60%) and the remaining 9 had advancing disease (response rate of 30%). At the end of the trial period, 8 of the 30 patients had passed away, 22 had survived (one patient had lost contact), 7 were still getting immune checkpoint inhibitors for maintenance and 14 patients had survived but were not receiving on-going medication but had stable disease upon follow-up. The most prominent limitations of this investigation were due to the small sample size, the diversity of patients with regard to underlying malignancy coupled with histology, the retrospective technique, which introduces much choice and recollection bias. To make these inferences stronger, prospective validation is needed.

## Conclusion:

Immune checkpoint inhibitors have shown remarkable efficacy in the treatment of metastatic lung cancer, particularly in patients with non-small cell lung cancer (NSCLC). These therapies target the PD-1/PD-L1 pathway and provide significant improvements for overall survival and progression-free survival by offering a new avenue for patients who previously had limited treatment options. However, immune-related adverse events (irAEs) can occur, ranging from mild to severe and require careful monitoring and management. While these inhibitors represent a promising advancement, the decision to use them must be individualized, considering factors such as patient health status, tumor characteristics and potential for adverse reactions. On-going research into biomarkers for predicting response and strategies for managing side effects will continue to refine their use, aiming to maximize their therapeutic benefit while minimizing harm.

## Figures and Tables

**Table 1:** Distribution of all parameter

**Parameters**		**Frequency (n)**	**Percent (%)**
Comorbidity	DM	4	13.3
	HTN	7	23.3
	DM, HTN	10	33.3
	HTN, DM, IHD	2	6.7
	Nil	7	23.3
	Total	30	100
Diagnosis	Nasopharynx	2	6.7
	NSCLC	19	63.3
	RCC	6	20
	Urinary Bladder	3	10
	Total	30	100
Histology	Adenocarcinoma	19	63.3
	Clear Cell Carcinoma Kidney	6	20
	Squamous cell carcinoma (SCC)	2	6.7
	Urothelial carcinoma	3	10
Metastatic Site	Bone	2	6.7
	Liver	9	30
	Brain, Bone	1	3.3
	Bone, Lymph node	1	3.3
	Liver, Bone	3	10
	Liver, Brain	2	6.7
	Liver, Lung	2	6.7
	Lung, Bone	3	10
	Liver, Lung, Bone	3	10
	Liver, Bone, Brain	2	6.7
	Lung, Bone, Brain	2	6.7
	Total	30	100
